# Impact of a multidomain lifestyle intervention on white matter integrity: the SUPERBRAIN exploratory sub-study

**DOI:** 10.3389/fnagi.2023.1242295

**Published:** 2023-09-20

**Authors:** Sun Min Lee, Sohui Kim, Jee Hyang Jeong, Chang Hyung Hong, Yoo Kyoung Park, Hae Ri Na, Hong-Sun Song, Hee Kyung Park, Muncheong Choi, Buong-O Chun, Seong Hye Choi, Jong-Min Lee, So Young Moon

**Affiliations:** ^1^Department of Neurology, Ajou University School of Medicine, Suwon, Republic of Korea; ^2^Department of Electronic Engineering, Hanyang University, Seoul, Republic of Korea; ^3^Department of Neurology, Ewha Womans University School of Medicine, Seoul, Republic of Korea; ^4^Department of Psychiatry, Ajou University School of Medicine, Suwon, Republic of Korea; ^5^Department of Medical Nutrition, Graduate School of East-West Medical Nutrition, Kyung Hee University, Suwon, Republic of Korea; ^6^Department of Neurology, Bobath Memorial Hospital, Seongnam, Republic of Korea; ^7^Department of Sports Sciences, Korea Institute of Sports Science, Seoul, Republic of Korea; ^8^Division of Psychiatry, University College London, London, United Kingdom; ^9^Exercowork, Seoul, Republic of Korea; ^10^Graduate School of Physical Education, College of Arts and Physical Education, Myongji University, Yongin, Republic of Korea; ^11^Department of Neurology, Inha University School of Medicine, Incheon, Republic of Korea; ^12^Department of Biomedical Engineering, Hanyang University, Seoul, Republic of Korea

**Keywords:** white matter integrity, cingulum cingulate gyrus, dementia, prevention, lifestyle, intervention, cognition, SUPERBRAIN

## Abstract

In the South Korean study to prevent cognitive impairment and protect BRAIN health through lifestyle intervention in at-risk elderly people (SUPERBRAIN), we evaluated the impact of a 24-week facility-based multidomain intervention (FMI) and home-based MI (HMI) on white matter integrity. Among 152 participants, aged 60–79 years without dementia but with ≥1 modifiable dementia risk factor, 19 FMI, 20 HMI, and 16 controls underwent brain MRI at baseline and 24 weeks. Between the intervention and control groups, we compared changes in fractional anisotropy (FA), mean diffusivity (MD), axial diffusivity (AD) and radial diffusivity (RD) at regions-of-interest (ROI) including the cingulum cingulate gyrus (CgC), cingulum hippocampus (CgH), superior longitudinal fasciculus (SLF), as well as the uncinate fasciculus (UF). In addition, correlations between total and standard scores cognitive domains of the Repeatable Battery for the Assessment of Neuropsychological Status (RBANS) or serum brain-derived neurotrophic factor (BDNF) and changes in brain image measures were evaluated at a statistical significance level of *p* < 0.05 (uncorrected for multiple corrections). The FA, MD, AD, and RD at each ROI at the baseline were not different among groups after Bonferroni correction. In the statistical analysis using two-way repeated measures ANOVA, any significant difference in longitudinal changes in the FA, MD, AD, and RD was not revealed. The statistical analysis, among the significant regions in paired *t*-test of the intervention group, compared with the control group, the FMI, HMI, and intervention group yielded significantly more beneficial effects on the AD of the CgC. In addition, longitudinal AD changes of the left CgC correlated with the BDNF changes (*r* = 0.280, *p* = 0.048). In this study, enhanced cognitive reserve after the multidomain lifestyle intervention could be revealed by changes in brain imaging for white matter integrity.

## 1. Introduction

Recent research suggests that multidomain lifestyle interventions, which encompass dietary counseling, physical exercise, cognitive training, and vascular/metabolic risk monitoring, can confer cognitive benefits to individuals at risk of developing cognitive decline. The Finnish Geriatric Intervention Study to Prevent Cognitive Impairment and Disability (FINGER) (Ngandu et al., 2015), exploratory subgroup analyses in the French Multidomain Alzheimer Preventive Trial (MAPT) (Andrieu et al., 2017; Chhetri et al., 2018), the Dutch Prevention of Dementia by Intensive Vascular Care (PreDIVA) (Moll van Charante et al., 2016) studies, or the South Korean study to prevent cognitive impairment and protect BRAIN health through lifestyle intervention in at-risk older adults (SUPERBRAIN) (Moon et al., 2021) have reported the cognitive benefits of a multidomain lifestyle intervention in participants with increased risk of dementia. It is suggested that these interventions may enhance cognitive reserve and reduce inflammation and vascular/oxidative damage in the brain.

Cognitive reserve refers to the brain’s efficiency in performing its functions and is determined by factors such as brain volume, cerebral metabolism, and neural network density. The efficacy of multidomain lifestyle interventions in enhancing cognitive reserve can be evaluated through changes in structural or functional brain imaging or neurotrophic factor levels, such as brain-derived neurotrophic factor (BDNF). A previous exploratory analysis of the SUPERBRAIN study showed that facility-based multidomain interventions (FMI) resulted in a significant increase in serum BDNF levels compared to the control group (Moon et al., 2021). However, the impact of multidomain lifestyle interventions on structural or functional brain imaging remains controversial. The findings from the SUPERBRAIN study indicate that a 24-week FMI can increase both global and regional cortical thickness (Moon et al., 2022a) and alter regional homogeneity in the resting-state functional brain magnetic resonance imaging (MRI) (Moon et al., 2022b). However, an exploratory MRI sub-study of the FINGER trial did not reveal any significant differences in regional brain volumes, cortical thickness, or white matter lesion volume between the intervention and control groups after 2 years in at-risk elderly individuals without substantial impairment (Stephen et al., 2019). The controversial results of the previous studies may have arisen due to the difference of their sample size and intervention durations. Trials with the smaller sample sizes (*N* < 160 participants) and shorter intervention durations (up to 24 weeks) are more likely to report intervention benefits on overall cognition, specific domains (e.g., spatial working memory, executive functioning), or biomarkers (Solomon et al., 2021). However, interestingly, another FINGER MRI sub-study that used diffusion tensor imaging (DTI) found that the intervention group had a greater decrease in fractional anisotropy (FA) than the control group, although no significant intergroup differences were observed in other diffusion parameters (Stephen et al., 2020).

In this study, we evaluated the impact of a 6-month multidomain lifestyle intervention on changes in white matter integrity of DTI using data from the SUPERBRAIN.

## 2. Methods

### 2.1. Study population

**Figure 1:**
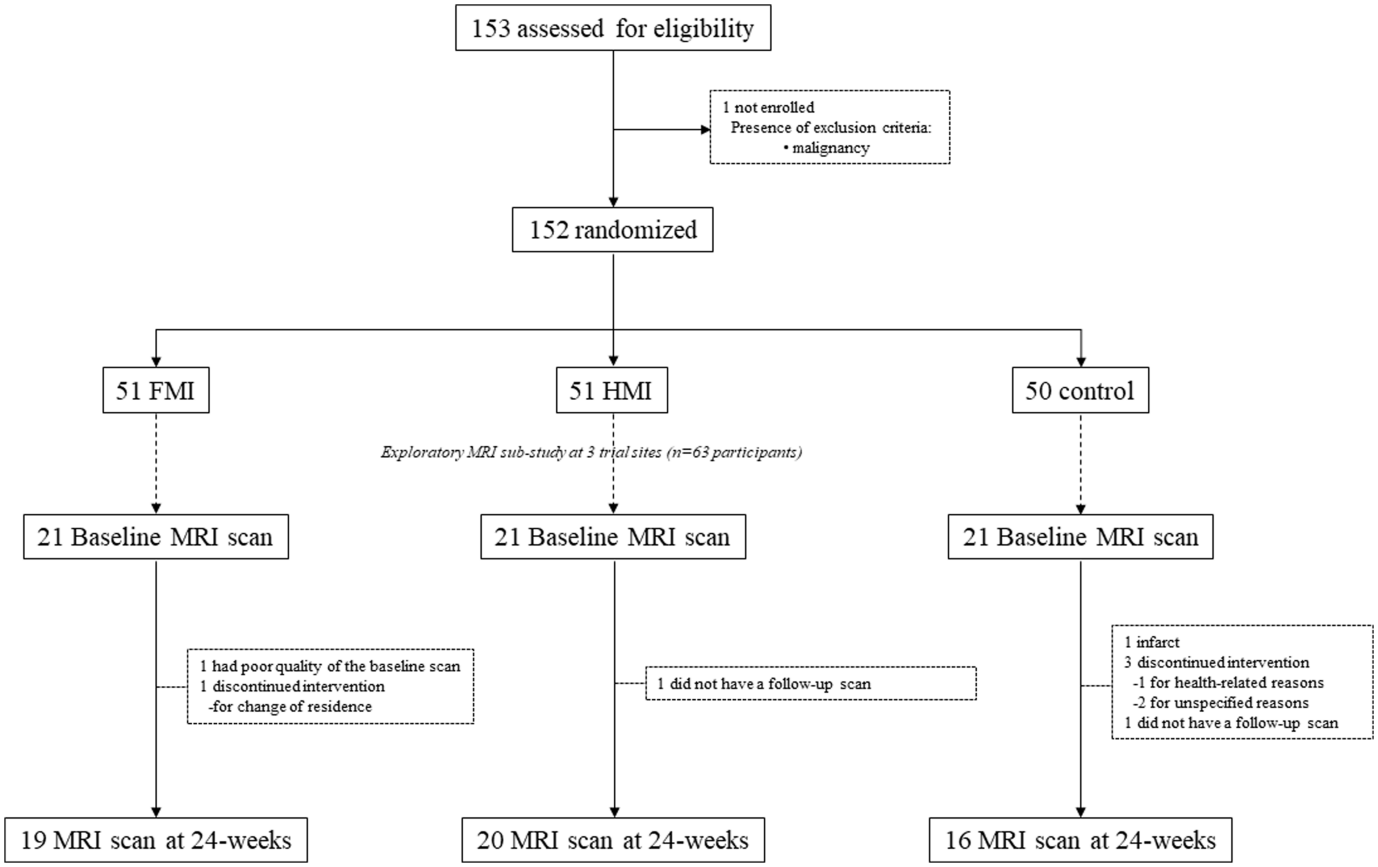
Diagram depicting the exploratory MRI analyses in the SUPERBRAIN trial. SUPERBRAIN, South Korean Study to Prevent Cognitive Impairment and Protect Brain Health Through Lifestyle Intervention; MRI, magnetic resonance imaging; FMI, facility-based multidomain intervention; HMI, home-based multidomain intervention.

The SUPERBRAIN trial protocol (ClinicalTrials.gov: NCT03980392) (Park et al., 2020) and primary findings (Moon et al., 2021) have been described previously. Briefly, this study was a 24-week randomized controlled trial conducted at three hospitals and five public health centers across South Korea, with a three-parallel-arm design including the FMI, home-based multidomain intervention (HMI), and control groups. Participants were selected from people who visited outpatient clinics or public health centers for memory problems, and those recruited through advertising. The study included 152 participants aged 60–79 years who had no dementia but had one or more modifiable dementia risk factors such as hypertension, diabetes mellitus, dyslipidemia, smoking, obesity, abdominal obesity, metabolic syndrome, educational level of ≤9 years, social isolation, and physical inactivity. In addition, they had a Mini-Mental State Examination *z* score of ≥ −1.5 based on the means and standard deviations in the age- and education-matched normal elderly Korean population (Han et al., 2008), were able to perform independent activities of daily living and pass a literacy test, and had a reliable informant who can provide investigators with the requested information. The individuals with major psychiatric illness such as major depressive disorder, dementia, Parkinson’s disease, malignancy within the previous 5 years, cardiac stent or revascularization within the previous 1 year, serious or unstable cardiovascular disease, other serious or unstable medical disease such as acute or severe asthma, active gastric ulcer, severe liver disease, or severe renal disease, as well as severe loss of vision, hearing, or communication disability were excluded. The study was conducted in accordance with the International Conference on Harmonization Good Clinical Practice Guidelines, and the institutional review boards of all institutions approved the protocol and consent forms before the start of the study. Written informed consent was obtained from all potential participants prior to enrollment. The SUPERBRAIN MRI exploratory sub-study included 63 participants from three trial sites (Ajou University Hospital, Ewha Womans University Medical Center, and Inha University Hospital) (Figure 1). Brain scans were conducted at baseline and 24-week visits. The present study included 55 participants with both baseline and repeat scans of good quality.

### 2.2. Randomization and intervention

The participants were randomly assigned to the FMI, HMI, and control groups at baseline at a 1:1:1 ratio. The FMI and HMI groups received interventions consisting of five components, including monitoring and management of metabolic and vascular risk factors, cognitive training and social activity, physical exercise (Lee et al., 2020), nutritional guidance, and motivational enhancement, as previously described in detail (Park et al., 2020; Moon et al., 2021).

Before the intervention, metabolic and vascular risk factors were assessed using blood tests and anthropometric measurements. These risk factors included hypertension, diabetes mellitus, dyslipidemia, obesity, abdominal obesity, smoking, and high alcohol consumption, which were monitored and managed. Study doctors met with each participant at baseline and week 12 to inform them of their risk factors and prescribe medications if necessary. Participants were educated about their risk factors and given lifestyle guidelines to prevent dementia by a study nurse at baseline. The nurse also met with participants every 4 weeks for measurements and monitoring of smoking and alcohol consumption. Measurements were recorded in the participants’ SUPERBRAIN notebooks. If a participant’s risk factors did not improve, they were re-educated by the nurse at week 12 using the booklets. The FMI group received facility-based interventions three times a week, while the HMI group received the same cognitive training and physical exercise programs as the FMI group but with a different schedule. In the first 2 months of the trial, the HMI group participated in one group-based cognitive training session (50 min) and one home-based cognitive training session (30–40 min) per week, and one group exercise session (60 min) and two home-based exercise sessions (60 min) per week. For the rest of the 6-month study, the HMI group attended one group cognitive training session and one group exercise session every 2 weeks. On weeks that included group sessions, the participants attended one cognitive training session and two exercise sessions individually at home each week. On weeks that did not include group sessions, participants attended two cognitive training sessions and three exercise sessions alone at home each week. The cognitive training focused on episodic memory, executive function, attention, working memory, calculation, and visuospatial function and was conducted using a tablet-based application or workbooks supervised by trained health professionals. The physical exercise program was designed to include aerobic exercise, exercises to enhance balance and flexibility, muscle-strengthening activities involving major muscle groups, and finger-and-toe movements. Trained exercise professionals guided the exercise programs during group sessions at a gym, using portable tools such as elastic bands, floor plates with numbers, and chairs. The exercise intensity increased every 2 months. During home-based exercise sessions, participants exercised by watching videos on a tablet PC or by following instructions in a poster or booklet. The nutritional intervention consisted of three individual counseling sessions (each lasting 30 min) and seven group sessions (each lasting 50 min) led by study nutritionists. The individual sessions involved tailoring the participant’s daily diet and providing education on customized diets to manage individual vascular risk factors. The group sessions provided discussions, practical exercises, and cooking lessons on making meals with recommended ingredients. Motivational enhancement included four group counseling sessions (each lasting 50 min) led by a psychologist at 1, 2, 13, and 24 weeks. These sessions provided information and support to facilitate dementia prevention program activities and included discussions on the importance of change, ambivalence, and self-efficacy, as well as family education using video clips. The family-coach program involved a family member in reinforcing the motivation of a participant. Encouraging pop-up video messages made by participants’ families and self-rated achievement pop-up messages were provided every week before the tablet-based cognitive intervention. Participants in workbook-type cognitive interventions were sent the encouraging pop-up video messages on their cell phones and performed self-rated achievement assessments on paper. The control group received regular health advice according to established guidelines.

### 2.3. Cognitive outcomes and serum BDNF

The study used the Repeatable Battery for the Assessment of Neuropsychological Status (RBANS) to evaluate cognitive function. The RBANS is a well-validated and reliable cognitive screening battery comprising 12 subtests that can be administered in 20–30 min with 4 alternate versions (A, B, C, and D) for reevaluation (Randolph et al., 1998). The subtests for each cognitive domain are as follows: digit span and coding for attention, picture naming and semantic fluency for language, figure copy and line orientation for visuospatial-construction, list learning and story memory for immediate memory, and list recall, list recognition, story recall and figure recall for delayed memory. Participants completed versions A and D at baseline and post-intervention, respectively, and a standard score was obtained for each cognitive domain based on same-aged peers. The total scale index score of cognitive functioning was calculated by combining these scores. Assessors received education on outcome measurements prior to the study (Park et al., 2020). Higher scores on the RBANS indicate better cognitive functioning. The assessment was conducted within 4 weeks post-intervention.

Changes in serum BDNF levels were investigated after the multidomain intervention. Fasting blood samples were collected at approximately 9 am using serum separator tubes (SSTs) within 4 weeks before and after the intervention. The SSTs were kept at room temperature for 30 min, centrifuged for 10 min at 3000 rpm, and sent to a central laboratory. Serum samples were stored at −70°C or lower until analysis. Serum BDNF levels were measured using a quantitative sandwich enzyme-linked immunosorbent assay (ELISA) (DBD00; R&D Systems, Inc., Minneapolis, MN, USA) in accordance with the manufacturer’s instructions.

### 2.4. MRI acquisition and processing

Diffusion-weighted MRI data at each clinical site were acquired with the following MR systems and DTI parameters: 3 T Achieva, Philips with a 8-channel SENSE head coil [32 diffusion sampling directions; *b*-value, 1,000 $s/mm^{2}$ ; 256 × 256 acquisition matrix with 80 slices; repetition time (TR), 11.659 s; echo time (TE), 75 ms; flip angle, 90°; voxel size, 0.89 × 0.89 × 2 $mm^{3}$ ; field of view, 230 mm ] at the Ajou University Hospital, 3 T Achieva, Philips with a 8-channel SENSE head coil (32 diffusion sampling directions; b-value, 1,000 $s/mm^{2}$ ; 128 × 128 acquisition matrix with 80 slices; TR, 11.659 s; TE, 92.222 ms; flip angle, 90°; voxel size, 1.79 × 1.79 × 2 $mm^{3}$ ; field of view, 230 mm ) at the Ewha Woman’s University Medical Center, and 3 T Signa Architect, GE with a 34-channel array head coil, at the Inha University Hospital (30 diffusion sampling directions; *b*-value, 1,000 $s/mm^{2}$ ; 256 × 256 acquisition matrix with 70 slices; TR, 15 s; TE, 79.371 ms; flip angle, 90°; voxel size, 0.89 × 0.89 × 2 $mm^{3}$ ; field of view, 230 mm ). The same imaging parameters and MRI scanners were used for the baseline and 24-week scans. Regular phantom scans were performed within each site, although the phantom was not shared among three centers.

**Figure 2:**
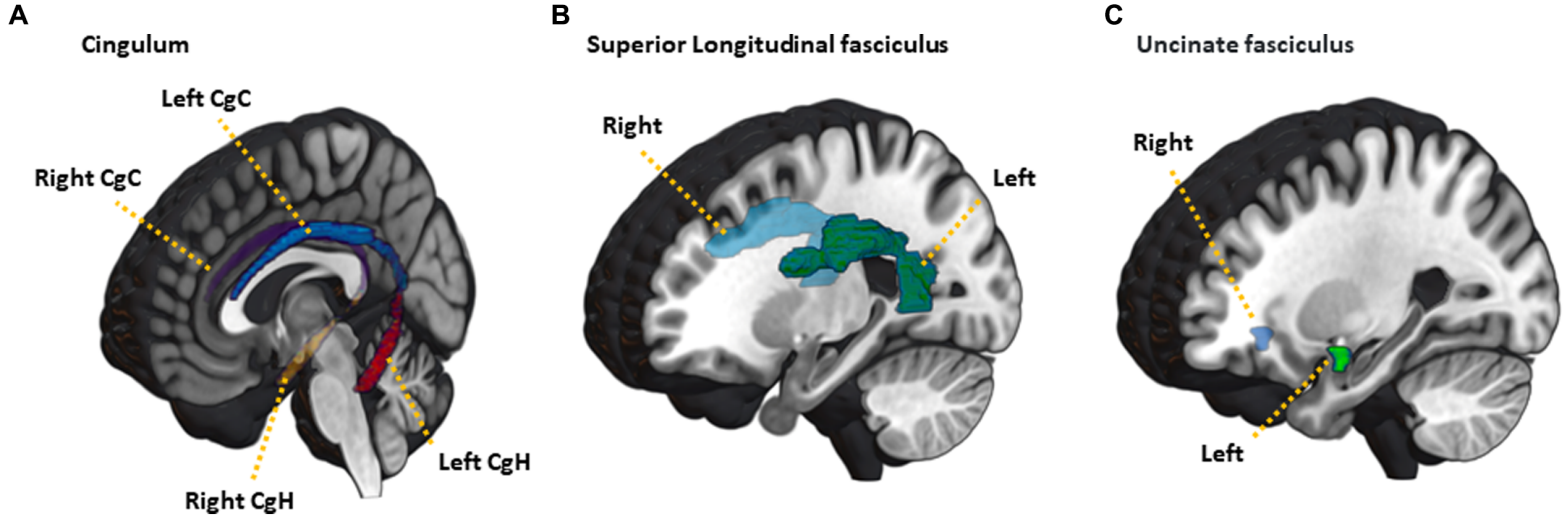
Illustration of JHU-ICBM-labels atlas ROIs used in the analysis. **(A)** cingulum, **(B)** superior longitudinal fasciculus, **(C)** uncinated fasciculus. CgC, cingulum cingulate gyrus; CgH, cingulum hippocampus.

The diffusion-weighted images were processed using FMRIB’s Software Library (FSL).^1^ The Brain Extraction Tool (BET) was used to remove skull and non-brain tissue to extract non-diffusion-weighted volume (b0 volume) brain regions. The EDDY tool^2^ (Andersson and Sotiropoulos, 2016) was used to estimate and correct for susceptibility-induced off-resonance field, volume movement, and eddy current distortions. The low-frequency MR intensity inhomogeneity and its effects on the diffusion images were estimated for the b0 volume and the estimated bias field map was applied to all diffusion-weighted volumes using the N4BiasFieldCorrection tool from Advanced Normalization Tools (ANTs) (Tustison et al., 2010). To correct for geometric distortions due to MR susceptibility in the diffusion data, previous studies have used additional diffusion-weighted images with reversed phase-encoding direction using the TOPUP tool^3^ (Andersson et al., 2003). However, most diffusion-weighted studies struggle to apply the TOPUP tool due to a lack of reversed data (Wang et al., 2017). Alternatively, nonlinear registration for a structural T1 MRI was performed to correct for distortions using symmetric regularization (SyN) implemented in ANTs (Avants et al., 2008). The bias-corrected and skull-stripped T1 MRI was inverted prior to non-linear registration for the similar contrast characteristics of the source and the target images. The dtifit tool was used to fit the diffusion tensor model and computed diffusion scalar maps for FA, mean diffusivity (MD), axial diffusivity (AD) and radial diffusivity (RD) using the tensor eigenvalues.^4^ Finally, we performed feature extraction by aligning JHU-ICBM-labels atlas to the native FA image using a SyN and calculating the average FA, MD, AD, and RD values for each region of interest (ROI) (Mori et al., 2005). Based on the results of previous imaging analyses from the SUPERBRAIN studies (Moon et al., 2022a,b), specific ROIs were selected where changes in white matter integrity were anticipated. These ROIs consisted of the cingulum cingulate gyrus (CgC), cingulum hippocampus (CgH), superior longitudinal fasciculus (SLF), as well as the uncinate fasciculus (UF) on both hemispheres (Figure 2).

### 2.5. Statistical analysis

Group differences at baseline were analyzed by *t*-test (MRI vs. non-MRI groups) or one-way analyses of variance (ANOVA, FMI vs. HMI vs. controls) for continuous variables and the chi-square test for categorical variables. In addition, two-way repeated measures ANOVA, with time and groups as independent factors, was used to compare changes in the RBANS and serum BDNF levels from baseline to the study endpoint. The groups were considered similar when *p* > 0.05. *Post hoc* analyses were done by Dunnett test.

Individual FA, MD, AD, and RD at baseline were compared among groups using one-way analyses of covariance (ANCOVA) with age, sex, years of education, and imaging site effects added as covariates of no interest. In addition, to compare changes in the FA, MD, AD, and RD from baseline to the study endpoint, we used two statistical analyses. First, two-way repeated measures ANOVA was used, with time and groups as independent factors and age, sex, years of education, and imaging site effects added as covariates of no interest. Secondly, ROI-wise paired *t*-tests were used to analyze longitudinal changes in white matter integrity for each group (FMI, HMI or controls) and combined intervention groups (FMI + HMI), respectively, using age, sex, years of education, and site effects as covariates. The statistical significance level was set at Bonferroni corrected *value of p* < 0.05. Significant brain image measures in previous paired *t*-test were compared between each group pair (FMI vs. control; HMI vs. control; FMI + HMI vs. control) using ANCOVA with changes as dependent variables and age, sex, education, and site as covariates. Finally, correlations between clinical parameters (total and standard scores cognitive domains of the RBANS) or the serum BDNF level and changes in significant brain image measures were evaluated after controlling for age, sex, and years of education at a statistical significance level of *p* < 0.05 (uncorrected for multiple corrections). Specifically, the statistical analyses were conducted using statsmodels.api.OLS package.^5^

## 3. Results

The population undergoing MRI was younger, more educated, and had a higher baseline RBANS total scale index than the population not undergoing MRI in SUPERBRAIN. However, there were no differences in intervention adherence, sex distribution, APOE ε4 carriers, and group distribution between the two populations (Supplementary Table S1). The intervention and control groups in the SUPERBRAIN exploratory MRI sub-study were not significantly different in demographic, clinical, cognitive, and MRI characteristics at baseline (Table 1). The adherence rates in the FMI and HMI groups were 96.0 and 97.0%, respectively.

**Table 1:** Baseline clinical characteristics in the intervention and control groups.

	FMI (*n* = 19)	HMI (*n* = 20)	Control (*n* = 16)	Value of *p*
Age, y	68.0 ± 4.7	68.8 ± 4.7	67.3 ± 4.4	0.657
Education, y	12.1 ± 3.8	11.6 ± 4.0	9.8 ± 4.3	0.314
Female, n (%)	13 (68.4)	12 (60.0)	14 (87.5)	0.314
APOE ε4 carriers, *n* (%)	3 (15.7)	2 (10.0)	3 (18.8)	0.724
RBANS indexes				
Total	119.9 ± 13.6	109.6 ± 19.1	105.5 ± 18.8	0.672
Attention	112.3 ± 14.1	112.3 ± 16.5	104.8 ± 18.1	0.257
Immediate recall	104.7 ± 16.2	106.1 ± 15.7	101.1 ± 15.2	0.658
Delayed recall	98.4 ± 11.6	99.7 ± 18.3	99.9 ± 17.4	0.942
Visuospatial/Construction	99.3 ± 11.2	101.5 ± 14.2	95.6 ± 13.4	0.433
Language	108.6 ± 15.1	111.8 ± 10.3	111.2 ± 15.1	0.769
BDNF, ng/ml	29.8 ± 11.3	29.9 ± 11.4	40.5 ± 22.1	0.158

FMI, facility-based multidomain intervention; HMI, home-based multidomain intervention; RBANS, Repeatable Battery for the Assessment of Neuropsychological Status; BDNF, brain-derived neurotrophic factor. Data for continuous variables are presented as the mean ± standard deviation. Group differences were analyzed by one-way analyses of variance (ANOVA) for continuous variables and the chi-square test for categorical variables. *P*-values represent the results from the ANOVA or chi-square test for the comparison of three groups (FMI vs HMI vs Control).

The two-way repeated measures ANOVA showed significant group × time interaction in the RBANS total scale index scores, delayed recall and visuospatial/construction (Table 2). *Post hoc* analyses revealed that longitudinal changes in the visuospatial/construction significantly differed between the FMI group and the control (*p* = 0.039) or between the HMI group and the control (*p* = 0.042). Serum BDNF levels did not show any significant group × time interaction among groups (Table 2).

**Table 2:** Changes in clinical characteristics after 24 weeks in the intervention and control groups.

	FMI (*n* = 19)	HMI (*n* = 20)	Control (*n* = 16)	*P*-value		
				Group	Time	Group×time
RBANS indexes						
Total	7.0 ± 6.3	5.2 ± 9.7	−2.3 ± 9.8	0.195	0.007	0.005
Attention	1.4 ± 8.1	−0.5 ± 9.0	−2.7 ± 9.1	0.085	0.683	0.357
Immediate recall	6.1 ± 9.3	7.3 ± 14.1	3.3 ± 10.4	0.401	0.001	0.702
Delayed recall	10.5 ± 10.8	11.0 ± 9.3	2.9 ± 7.9	0.732	<0.001	0.027
Visuospatial/Construction	3.0 ± 9.3	−0.9 ± 16.4	−8.4 ± 13.4	0.035	0.200	0.044
Language	2.0 ± 12.0	0.1 ± 11.5	−3.1 ± 11.1	0.873	0.947	0.391
BDNF, ng/ml	14.6 ± 24.5	4.2 ± 2.2	−3.7 ± 2.6	0.750	0.099	0.088

FMI, facility-based multidomain intervention; HMI, home-based multidomain intervention, RBANS, Repeatable Battery for the Assessment of Neuropsychological Status; BDNF, brain-derived neurotrophic factor. The two-way repeated measures one-way analyses of variance (ANOVA), with time and groups as independent factors, was used to compare changes in the RBANS and serum BDNF levels from baseline to the study endpoint. *P*-values represent the results from the two-way repeated measures ANOVA to exam groups (FMI vs HMI vs Control) × time (baseline vs. post-intervention) effects.

### 3.1. Longitudinal changes in FA, MD, AD, and RD

**Figure 3:**
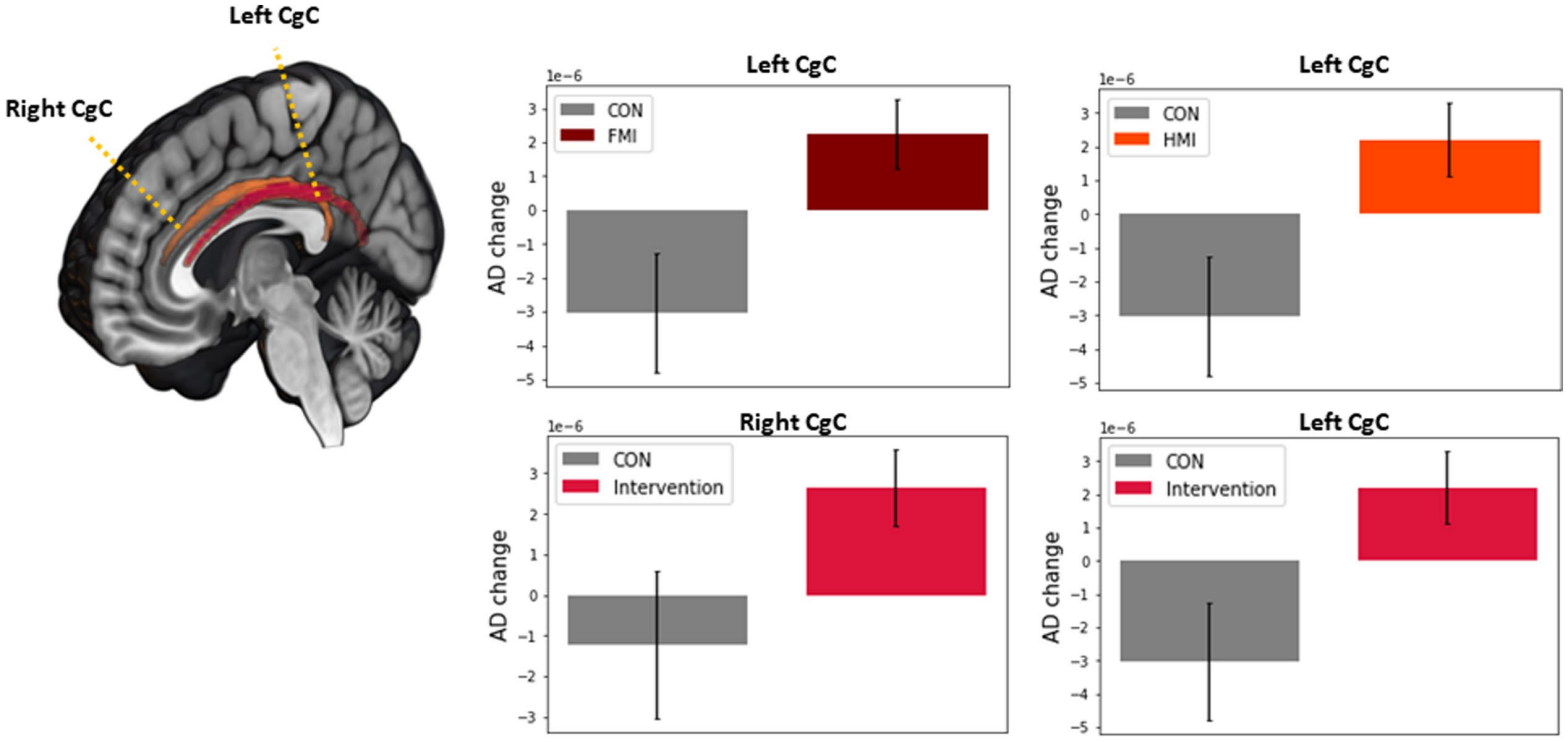
Comparison between intervention group and the control of white matter integrity in the regions with significant longitudinal change in the intervention group. Among the significant regions in paired *t*-test of the intervention group, compared with the control group, the FMI, HMI, and intervention group yielded significantly more beneficial effects on the AD of the CgC. AD, axial diffusivity; CgC, cingulum cingulate gyrus; FMI, facility-based multidomain intervention; HMI, home-based multidomain intervention; CON, control.

The FA, MD, AD, and RD at each ROI at the baseline were not different among groups after Bonferroni correction. In the first statistical analysis using two-way repeated measures ANOVA, any significant difference in longitudinal changes in the FA, MD, AD, and RD was not revealed. The second statistical analysis, which analyzed longitudinal changes in white matter integrity for each group, revealed the results as follows: FA increase (*p-*value = 0.0003, *t* value = −4.625) and RD decrease (*p-*value = 0.0026, *t* value = 3.599) of the left UF in the control group; MD decrease (*p-*value = 0.0061, *t* value = 3.106) of the left CgH in the FMI; AD decrease (*p-*value = 0.0009, *t* value = 3.939) of the right CgH in the HMI. However, the difference in these longitudinal changes between groups was not significant. Additionally, in the combined intervention groups (FMI + HMI), ROI-wise paired *t*-tests showed significant longitudinal changes in white matter integrity of the cingula and right UF (Table 3). Among the significant regions in paired *t*-test of the combined intervention groups, compared with the control group, the FMI, HMI, and intervention group yielded significantly more beneficial effects on the AD of the CgC (Table 4; Figure 3).

**Table 3:** Longitudinal changes between baseline and post-intervention in white matter integrity in each group.

	Baseline				Changes (from baselines to study end)				*value of p*			
	FMI	HMI	FMI + HMI	Control	FMI	HMI	FMI + HMI	Control	FMI	HMI	FMI + HMI	Control
	(*n* = 19)	(*n* = 20)	(*n* = 39)	(*n* = 16)	(*n* = 19)	(*n* = 20)	(*n* = 39)	(*n* = 16)				
**FA**												
CgC, right	0.0980(0.0075)	0.1013(0.0076)	0.0999(0.0077)	0.1008(0.0064)	0.0005(0.0047)	−0.0002(0.0047)	0.0001(0.0047)	−0.0001(0.0052)	0.6336	0.7831	0.892	0.8926
CgC, Left	0.1029(0.0072)	0.1071(0.0096)	0.1054(0.0089)	0.1054(0.0071)	0.0008(0.0052)	−0.0002(0.0054)	0.0002(0.0053)	−0.0001(0.0062)	0.5146	0.8657	0.7409	0.9385
CgH, right	0.1183(0.0096)	0.1202(0.0086)	0.1193(0.0089)	0.1174(0.0108)	0.0051(0.0102)	0.0033(0.0124)	0.0041(0.0113)	0.0011(0.0106)	0.424	0.2505	0.0563	0.6796
CgH, Left	0.1085(0.0100)	0.1121(0.0103)	0.1106(0.0102)	0.1069(0.0090)	0.0056(0.0101)	0.0042(0.0108)	0.0049(0.0103)	0.0061(0.0121)	0.0516	0.0966	***0.0051***	0.0611
SLF, right	0.1179(0.0075)	0.1248(0.0091)	0.1215(0.0088)	0.1227(0.0079)	0.0007(0.0040)	−0.0001(0.0055)	0.0002(0.0048)	0.0002(0.0051)	0.4534	0.9096	0.7284	0.8533
SLF, left	0.1154(0.0056)	0.1218(0.0082)	0.1189(0.0079)	0.1196(0.0056)	0.0008(0.0040)	0.0008(0.0044)	0.0008(0.0041)	0.0005(0.0041)	0.3552	0.4204	0.2145	0.6028
UF, right	0.0893(0.0078)	0.0943(0.0080)	0.0922(0.0084)	0.0888(0.0080)	0.0008(0.0023)	−0.0001(0.0041)	0.0003(0.0034)	−4.26E-05(0.0050)	0.138	0.8606	0.5509	0.9736
UF, left	0.0753(0.0097)	0.0802(0.0068)	0.0779(0.0085)	0.0754(0.0080)	0.0011(0.0050)	0.0019(0.0050)	0.0015(0.0049)	0.0031(0.0026)	0.3152	0.1077	0.0581	***0.0003***
**MD**												
CgC, right	0.0001(4.78E-06)	0.0001(5.79E-06)	0.0001(5.44E-06)	0.0001(6.43E-06)	1.49E-06(4.28E-06)	1.67E-06(4.63E-06)	1.58E-06(4.41E-06)	−5.31E-07(6.93E-06)	0.1454	0.1219	0.0502	0.7633
CgC, Left	0.0001(6.08E-06)	0.0001(6.84E-06)	0.0001(6.68E-06)	0.0001(8.13E-06)	8.65E-07(4.49E-06)	1.31E-06(5.29E-06)	1.09E-06(4.86E-06)	−1.56E-06(6.42E-06)	0.4122	0.2801	0.167	0.3465
CgH, right	0.0001(1.01E-05)	0.0001(1.20E-05)	0.0001(1.09E-05)	0.0001(1.03E-05)	−9.13E-06(1.36E-05)	−5.80E-06(1.06E-05)	−7.42E-06(1.21E-05)	−3.54E-06(1.40E-05)	0.0514	0.051	***0.0004***	0.3295
CgH, Left	0.0001(1.24E-05)	0.0001(8.76E-06)	0.0001(1.11E-05)	0.0001(9.12E-06)	−1.07E-05(1.50E-05)	−6.36E-06(1.37E-05)	−8.50E-06(1.43E-05)	−6.49E-06(1.36E-05)	***0.0061***	0.0515	***0.0006***	0.0765
SLF, right	0.0001(7.67E-06)	0.0001(8.00E-06)	0.0001(7.92E-06)	0.0001(6.63E-06)	1.24E-06(4.00E-06)	1.53E-06(5.56E-06)	1.39E-06(4.80E-06)	1.12E-06(6.84E-06)	0.1919	0.2316	0.0777	0.5223
SLF, left	0.0001(1.12E-05)	0.0001(7.09E-06)	0.0001(9.91E-06)	0.0001(6.70E-06)	7.47E-07(5.74E-06)	1.24E-06(5.10E-06)	1.00E-06(5.35E-06)	−4.29E-07(4.69E-06)	0.5775	0.2872	0.2488	0.7192
UF, right	0.0001(4.43E-06)	0.0001(5.77E-06)	0.0001(5.30E-06)	0.0001(9.14E-06)	2.65E-06(6.86E-06)	3.33E-06(5.99E-06)	3.00E-06(6.35E-06)	2.74E-06(9.01E-06)	0.1083	0.0512	***0.0054***	0.2414
UF, left	0.0001(8.74E-06)	0.0001(1.04E-05)	0.0001(9.81E-06)	0.0001(7.59E-06)	−5.88E-07(4.08E-06)	1.11E-06(7.92E-06)	2.87E-07(6.32E-06)	−2.60E-06(4.54E-06)	0.5374	0.5348	0.7783	0.057
**AD**												
CgC, right	0.0002(9.00E-06)	0.0002(1.06E-05)	0.0002(9.64E-06)	0.0002(9.84E-06)	3.32E-06(5.33E-06)	2.64E-06(4.34E-06)	2.98E-06(4.80E-06)	−1.23E-06(7.53E-06)	0.0512	0.0534	***0.0004***	0.5211
CgC, Left	0.0002(1.12E-05)	0.0002(1.20E-05)	0.0002(1.13E-05)	0.0002(1.20E-05)	2.25E-06(4.62E-06)	2.21E-06(5.05E-06)	2.23E-06(4.78E-06)	−3.04E-06(7.30E-06)	0.0511	0.0648	***0.0059***	0.116
CgH, right	0.0002(1.34E-05)	0.0002(1.50E-05)	0.0002(1.40E-05)	0.0002(1.77E-05)	−1.18E-05(1.75E-05)	−7.45E-06(8.46E-06)	−9.61E-06(1.36E-05)	−6.03E-06(1.59E-05)	0.0502	***0.0009***	***<0.0001***	0.1503
CgH, Left	0.0002(2.60E-05)	0.0002(1.34E-05)	0.0002(2.05E-05)	0.0002(1.35E-05)	−1.40E-05(2.10E-05)	−7.43E-06(1.77E-05)	−1.06E-05(1.94E-05)	−5.05E-06(1.84E-05)	0.051	0.0766	***0.0015***	0.2909
SLF, right	0.0002(1.08E-05)	0.0002(1.17E-05)	0.0002(1.10E-05)	0.0002(8.60E-06)	2.48E-06(5.43E-06)	2.37E-06(5.93E-06)	2.42E-06(5.62E-06)	2.08E-06(7.41E-06)	0.0619	0.0898	0.0504	0.2783
SLF, left	0.0002(1.54E-05)	0.0002(8.72E-06)	0.0002(1.22E-05)	0.0002(8.88E-06)	1.74E-06(7.15E-06)	3.10E-06(6.31E-06)	2.44E-06(6.68E-06)	−2.96E-08(6.96E-06)	0.3016	0.0501	0.058	0.9866
UF, right	0.0002(1.09E-05)	0.0002(1.01E-05)	0.0002(1.04E-05)	0.0002(1.12E-05)	5.01E-06(1.04E-05)	5.60E-06(9.31E-06)	5.32E-06(9.75E-06)	4.74E-06(1.13E-05)	0.0507	0.0544	***0.0015***	0.1156
UF, left	0.0001(1.43E-05)	0.0001(1.38E-05)	0.0001(1.38E-05)	0.0001(1.34E-05)	8.26E-07(5.44E-06)	4.27E-06(1.03E-05)	2.59E-06(8.40E-06)	−6.20E-07(7.47E-06)	0.5167	0.0801	0.0612	0.7443
**RD**												
CgC, right	7.52E-05(6.57E-06)	7.22E-05(6.99E-06)	7.33E-05(7.08E-06)	7.31E-05(7.02E-06)	5.81E-07(5.28E-06)	1.19E-06(5.61E-06)	8.93E-07(5.39E-06)	−1.78E-07(7.42E-06)	0.6371	0.3551	0.3073	0.9244
CgC, Left	7.38E-05(7.02E-06)	6.98E-05(9.37E-06)	7.13E-05(8.79E-06)	7.17E-05(8.80E-06)	1.73E-07(5.81E-06)	8.69E-07(6.73E-06)	5.30E-07(6.23E-06)	−8.17E-07(7.70E-06)	0.898	0.5709	0.5983	0.6771
CgH, right	8.90E-05(1.10E-05)	8.61E-05(1.14E-05)	8.74E-05(1.10E-05)	8.78E-05(1.07E-05)	−7.76E-06(1.32E-05)	−4.97E-06(1.27E-05)	−6.33E-06(1.29E-05)	−2.29E-06(1.42E-05)	0.05	0.098	***0.004***	0.5274
CgH, Left	8.99E-05(9.93E-06)	8.45E-05(9.77E-06)	8.68E-05(1.03E-05)	8.95E-05(9.33E-06)	−9.11E-06(1.36E-05)	−5.83E-06(1.29E-05)	−7.42E-06(1.31E-05)	−7.21E-06(1.32E-05)	0.0511	0.0577	***0.0011***	0.056
SLF, right	0.0001(8.51E-06)	0.0001(9.44E-06)	0.0001(9.49E-06)	0.0001(8.40E-06)	6.28E-07(4.52E-06)	1.12E-06(6.43E-06)	8.80E-07(5.52E-06)	6.37E-07(7.10E-06)	0.5524	0.4456	0.3252	0.7245
SLF, left	0.0001(1.01E-05)	0.0001(9.25E-06)	0.0001(1.07E-05)	0.0001(7.36E-06)	2.47E-07(5.76E-06)	3.20E-07(5.38E-06)	2.84E-07(5.50E-06)	−6.30E-07(4.96E-06)	0.8534	0.7932	0.748	0.6188
UF, right	7.76E-05(6.67E-06)	7.24E-05(7.63E-06)	7.47E-05(7.69E-06)	7.77E-05(1.00E-05)	1.48E-06(5.50E-06)	2.19E-06(5.67E-06)	1.84E-06(5.53E-06)	1.75E-06(8.57E-06)	0.2566	0.1002	0.0539	0.4267
UF, left	7.55E-05(1.09E-05)	6.87E-05(1.02E-05)	7.19E-05(1.08E-05)	7.41E-05(8.50E-06)	−1.29E-06(5.63E-06)	−4.59E-07(7.76E-06)	−8.67E-07(6.73E-06)	−3.59E-06(3.99E-06)	0.3289	0.7943	0.4264	***0.0026***

FA, fractional anisotropy; MD, mean diffusivity; AD, axial diffusivity; RD, radial diffusivity; CgC, cingulum cingulate gyrus; CgH, cingulum hippocampus; SLF, superior longitudinal fasciculus; UF, uncinate fasciculus; FMI, facility-based multidomain intervention; HMI, home-based multidomain intervention. Data are presented as the mean (standard deviation). Regions of intertest-wise paired *t*-tests were used to analyze longitudinal changes in white matter integrity for each group (FMI, HMI or controls) and combined intervention groups (FMI + HMI), respectively, using age, sex, years of education, and site effects as covariates. The statistical significance level was set at Bonferroni corrected value of *p* < 0.05 (bolded and italicized). *P-*values represent the results from the paired *t*-tests in each group.

**Table 4:** Comparison between intervention group and the control of white matter integrity in the regions with significant longitudinal change in each group.

	Value of *p*		
	FMI vs. Control	HMI vs. Control	Intervention vs. Control
**FMI**			
**MD**			
CgH, Left	0.472	0.6674	0.9283
**HMI**			
**AD**			
CgH, right	0.4748	0.9627	0.6161
**FMI + HMI**			
**FA**			
CgH, Left	0.6149	0.4551	0.4961
**MD**			
CgH, right	0.2935	0.6842	0.3961
CgH, Left	0.492	0.6608	0.9288
UF, right	0.8694	0.9535	0.9764
**AD**			
CgC, right	0.1018	0.101	0.0469
CgC, Left	***0.0357***	***0.0352***	***0.0073***
CgH, right	0.4748	0.9627	0.6161
CgH, Left	0.235	0.9936	0.5641
UF, right	0.8323	0.747	0.8332
**RD**			
CgH, right	0.2535	0.5851	0.3472
CgH, Left	0.8827	0.4663	0.7453
**Controls**			
**FA**			
UF, left	0.1471	0.3877	0.1928
**RD**			
UF, left	0.2003	0.0902	0.0831

FA, fractional anisotropy; MD, mean diffusivity; AD, axial diffusivity; RD, radial diffusivity; CgC, cingulum cingulate gyrus; CgH, cingulum hippocampus; SLF, superior longitudinal fasciculus; UF, uncinate fasciculus; FMI, facility-based multidomain intervention; HMI, home-based multidomain intervention. Significant brain image measures in previous paired *t*-test in each group were compared between each group pair (FMI vs. control; HMI vs. control; FMI + HMI vs. control) using one-way analyses of covariance with changes as dependent variables and age, sex, education, and site as covariates. Significant *P-*value (*p* < 0.05) was bolded and italicized.

### 3.2. Correlations between AD of the CgC and clinical variables

Correlations between AD of the CgC and clinical variables were evaluated in participants. Although longitudinal AD changes of the CgC did not correlate significantly with changes in the RBANS, longitudinal AD changes of the left CgC correlated with the BDNF changes (*r* = 0.280, *p* = 0.048).

## 4. Discussion

Our study revealed that among the regions with significant longitudinal changes in the intervention group, compared with the control group, the FMI, HMI, and intervention group yielded significantly more beneficial effects on the AD of the CgC. In addition, longitudinal AD changes of the left CgC positively correlated with the BDNF changes. Therefore, in this study, enhanced cognitive reserve after the multidomain lifestyle intervention could be revealed by changes in brain imaging for white matter integrity.

Previous imaging analyses from the SUPERBRAIN studies (Moon et al., 2022a,b) have shown that group preventive strategies implemented at the facility can be beneficial in promoting structural or functional neuroplastic changes in brain areas that are involved in learning. These areas included the bilateral frontotemporal lobes, cingulate gyri, insula, and the left medial orbitofrontal gyrus. Based on these findings, we specifically selected the cingulum, SLF, and the UF on both hemispheres as ROIs where we expected to observe changes in white matter integrity. Among the chosen ROIs, our analyses demonstrated that the FMI, HMI, and intervention groups exhibited significantly more positive effects on the AD of the CgC compared to the control group. Furthermore, longitudinal AD changes in the left CgC were found to be positively correlated with changes in the BDNF. The cingulum bundle is one of the most distinctive fiber tracts in the brain, forming a near-complete ring from the orbital frontal cortices, along the dorsal surface of the corpus callosum, then down the temporal lobe towards the pole (Bubb et al., 2018). The cingulum is a complex pathway consisting of both short and long sagittal association fibers. Additionally, it contains fibers that extend across the tract, connecting cortical and subcortical regions. Among these sagittal connections, there are numerous short cortico-cortical association fibers, often referred to as “U-fibers,” which interconnect the medial regions of the frontal, parietal, and temporal lobes. Given the length and intricacy of the cingulum bundle, there are various ways to subdivide it. For our analysis, we employed the JHU-ICBM-labels atlas, which divides the cingulum into two subdivisions: the CgC and the CgH. Previous research has suggested that the CgC is associated with attention and executive functions, whereas the CgH is more closely related to learning and episodic memory. In our study, we found that longitudinal changes in the CgC did not significantly correlate with changes in the RBANS. However, it is worth highlighting that previous studies have shown that multidomain lifestyle interventions or exercise can have a positive impact on various cognitive domains, including global cognition, processing speed/attention, and executive function (Ngandu et al., 2015; Gomes-Osman et al., 2018). These cognitive functions are associated with the CgC. Therefore, it is possible that interventions targeting multidomain lifestyle or exercise could potentially affect the CgC and contribute to improvements in attention, processing speed, and executive function.

Several studies indicate that the effects of aging on the cingulum are not uniform and can vary across different regions. Evidence suggests an age-related gradient along the long axis of the cingulum, with the frontal parts of the bundle being most affected (Bubb et al., 2018). Conversely, consistent findings have shown microstructural changes in the posterior and parahippocampal cingulum, which are associated with both mild cognitive impairment and Alzheimer’s disease (Yu et al., 2017). In our study, we observed significantly more beneficial effects on the AD of the CgC in the FMI, HMI, and intervention groups. Furthermore, we found a positive correlation between longitudinal AD changes in the left CgC and changes in BDNF. The possible mechanisms of dementia protection by multidomain lifestyle intervention involve increasing or maintaining cognitive reserve in the presence of pathology and neuropathological damage (Livingston et al., 2017, 2020). These cognitive reserve mechanisms may include preserved metabolism or increased connectivity in temporal and frontal brain areas. A previous study has suggested that increased resilience against cognitive impairment in the early stages of Alzheimer’s disease is partially associated with enhanced connectivity between the left frontal cortex and brain hubs (Franzmeier et al., 2018). Building upon these findings, our study contributes further evidence regarding the mechanisms underlying dementia prevention through multidomain lifestyle interventions. Specifically, we observed improved white matter integrity in the CgC, providing support for the beneficial effects of such interventions on maintaining the structural integrity of this region. Our multidomain lifestyle intervention revealed only beneficial effects on the AD among various DTI parameters. AD refers to the magnitude of diffusion parallel to fiber tracts. Lower AD might reflect axonal injury, reduced axonal caliber, or less coherent orientation of axons. FA refers to the fraction of diffusion that is directionally dependent (anisotropic). Lower FA might reflect damage to the myelin sheath surrounding axons, enlarged axonal diameter, reduced axonal packing density, or increased membrane permeability. MD is the overall directionally averaged magnitude of diffusion, and its increase reflects reduced white matter integrity due to either axonal or myelin degradation. Finally, RD refers to the magnitude of diffusion perpendicular to fiber tracts. RD may be relatively more sensitive to myelin, but higher RD might reflect myelin loss, or loss of axons and/or reduced axonal packing density. Together, FA and MD provide information about changes to barriers to diffusion; increased AD has been associated with axonal degeneration and increased RD has been linked to demyelination (Bosch et al., 2012; Bennett and Madden, 2014; Kantarci, 2014; Alves et al., 2015). In addition, there is evidence that AD is not influenced by myelin. Therefore, the AD is differentiated from other DTI parameters in terms of its specific relation to the axonal change. The Amyloid Cascade Hypothesis (Hardy and Higgins, 1992) predicts that axonal degeneration is a result of Wallerian degeneration and precedes neuronal loss. The close association of tau with both axonal integrity and with the cognitive symptoms of Alzheimer’s disease suggests that white matter changes may occur independently and perhaps prior to changes in gray matter. Building upon these findings, our study contributes further evidence regarding the mechanisms underlying dementia prevention through multidomain lifestyle interventions. Specifically, we observed improved white matter integrity through the effect on the AD, providing support for the beneficial effects of such interventions on maintaining the structural integrity against the axonal degeneration rather than changes in the myelin integrity.

The main strengths of this study are the randomized controlled design, the multidomain intervention, and the availability of MRI scans at baseline and 6 month. The main limitation of this study is that the MRI scanners differed between sites; however, this was adjusted for during analysis. Additionally, as this was a SUPERBRAIN MRI exploratory sub-study, the results should be interpreted with caution. Future studies including white matter integrity as a primary outcome measure are necessary to confirm the impact of multidomain lifestyle interventions on white matter integrity.

In conclusion, significant changes in white matter integrity in at-risk elderly without substantial impairment after the multidomain lifestyle intervention suggest that multidomain lifestyle intervention may confer cognitive benefits through neuroplastic changes of functional processing circuits in the brain areas which play a crucial role in adaptive learning.

## Data availability statement

The raw data supporting the conclusions of this article will be made available by the authors, without undue reservation.

## Ethics statement

The studies involving humans were approved by Ajou University Hospital institutional review board. The studies were conducted in accordance with the local legislation and institutional requirements. The participants provided their written informed consent to participate in this study.

## Author contributions

SM, JJ, CH, YP, J-ML, and SC: conceptualization and methodology. SM and SK: formal analysis and investigation. SM, SL, and SK: writing – original draft preparation. SM, JJ, CH, YP, J-ML, and SC: writing – review and editing. SM, JJ, CH, YP, J-ML, and SC: funding acquisition. All authors contributed to manuscript revision, read, and approved the submitted version.

## Funding

This work was supported by grants from the National Research Council of Science & Technology (NST) Aging Convergence Research Center (CRC22011-600) and from Korea Health Technology R&D Project through the Korea Health Industry Development Institute (KHIDI) and Korea Dementia Research Center (KDRC), funded by the Ministry of Health & Welfare and Ministry of Science and ICT, Republic of Korea (HI18C0479, HU20C0198, and HU21C0016). The funders had no role in the study design, data collection, analysis, interpretation of data, writing of the report, or decision to submit the manuscript for publication.

## Conflict of interest

SM received a research grant from Hyundai Pharmaceutical Co. Ltd. CH receives research support from Eisai Korea Inc. JJ receives research grants from Chong Kun Dang Pharmaceutical Corp., Jeil Pharmaceutical Co. Ltd., and Kuhnil Pharmaceutical Co. Ltd., and consults for PeopleBio Co. Ltd. SM, CH, JJ, YP, HN, and SC are shareholders of Rowan Inc. YP consults for Pulmuone Co. Ltd. HN consults for Hyundai Pharmaceutical Co. Ltd. SC consults for Hyundai Pharmaceutical Co. Ltd. and PeopleBio Co. Ltd.

The remaining authors declare that the research was conducted in the absence of any commercial or financial relationships that could be construed as a potential conflict of interest.

## Publisher’s note

All claims expressed in this article are solely those of the authors and do not necessarily represent those of their affiliated organizations, or those of the publisher, the editors and the reviewers. Any product that may be evaluated in this article, or claim that may be made by its manufacturer, is not guaranteed or endorsed by the publisher.

## Footnotes

^1^http://www.fmrib.ox.ac.uk/fsl

^2^https://fsl.fmrib.ox.ac.uk/fsl/fslwiki/eddy

^3^https://fsl.fmrib.ox.ac.uk/fsl/fslwiki/topup

^4^https://fsl.fmrib.ox.ac.uk/fsl/fslwiki/dtifit

^5^https://www.statsmodels.org/dev/generated/statsmodels.regression.linear_model.OLS.html
