# Supplementary material for: Impact of a multidomain lifestyle intervention on white matter integrity: the SUPERBRAIN exploratory sub-study
Source: Front Aging Neurosci. 2023 Sep 20;15:1242295. doi: 10.3389/fnagi.2023.1242295 (PMC10548201; doi:10.3389/fnagi.2023.1242295)
Supplement: Supplementary file 1 [file Table_1.docx]

|  | **Total** | **MRI**  **(n=55)** | **No MRI**  **(n=97)** | **p** |
| --- | --- | --- | --- | --- |
| **Age, y** | 152 | 68.2±4.5 | 73.1±3.9 | <0.001 |
| **Education, y** | 152 | 11.1±4.0 | 9.3±5.1 | 0.015 |
| **Female, n (%)** | 152 | 38 (69.1) | 74 (76.3) | 0.344 |
| **APOE ε4 carriers, n (%)** | 151 | 8 (14.8) | 18 (18.6) | 0.656 |
| **RBANS at baseline** | 152 | 108.6±17.3 | 95.8±17.9 | <0.001 |
| **Group (control/FMI/HMI), n (%)** | 152 | 16 (29.1)/20 (36.4)/19 (34.5) | 34 (35.1)/31 (32.0)/32 (33.0) | 0.738 |
| **Adherence, %** | 102 | 96.5±4.6 | 95.1±10.1 | 0.426 |
